# Impaired baroreflex sensitivity and increased systolic blood pressure variability in chronic post-ischemic stroke

**DOI:** 10.6061/clinics/2018/e253

**Published:** 2018-09-24

**Authors:** Juliana Valente Francica Grilletti, Katia Bilhar Scapini, Nathalia Bernardes, Jaqueline Spadari, Aline Bigongiari, Flavia de Andrade e Souza Mazuchi, Erico Chagas Caperuto, Iris Callado Sanches, Bruno Rodrigues, Kátia De Angelis

**Affiliations:** I Laboratorio do Movimento Humano, Universidade Sao Judas Tadeu (USJT), Sao Paulo, SP, BR; II Laboratorio de Fisiologia Translacional, Universidade Nove de Julho (UNINOVE), Sao Paulo, SP, BR; III Universidade Sao Judas Tadeu (USJT), Sao Paulo, SP, BR; IV Departamento de Educacao Fisica Adaptada, Faculdade de Educacao Fisica, Universidade Estadual de Campinas (UNICAMP), Campinas, SP, BR; V Departamento de Fisiologia, Universidade Federal de Sao Paulo (UNIFESP), Sao Paulo, SP, BR

**Keywords:** Stroke, Autonomic Nervous System, Baroreflex

## Abstract

**OBJECTIVES:**

Acute post-stroke patients present cardiovascular autonomic dysfunction, which manifests as lower heart rate variability and impaired baroreflex sensitivity. However, few studies performed to date have evaluated cardiovascular autonomic function in chronic post-stroke patients. The aim of this study was to evaluate cardiovascular autonomic modulation in chronic post-ischemic stroke patients.

**METHODS:**

The seventeen enrolled subjects were divided into a stroke group (SG, n=10, 5±1 years after stroke) and a control group (CG, n=7). Non-invasive curves for blood pressure were continuously recorded (Finometer®) for 15 minutes while the subject was in a supine position. Heart rate variability and blood pressure variability were analyzed in the time and frequency domains.

**RESULTS:**

No differences were observed in systolic and diastolic pressure and heart rate between post-stroke patients and healthy individuals. The SG group had lower indexes for heart rate variability in the time domain (standard deviation of normal to normal R-R intervals, SDNN; variance of normal to normal R-R intervals, VarNN; and root mean square differences of successive R-R intervals, RMSSD) and a lower high-frequency band for heart rate variability than was observed in the CG. Systolic blood pressure variability and the low-frequency band for systolic pressure were higher in post-stroke patients, while the alpha index was lower in the SG than in the CG.

**CONCLUSION:**

After ischemic stroke, affected patients present chronically reduced heart rate variability, impaired cardiac vagal modulation, increased systolic blood pressure variability and higher sympathetic vascular modulation along with impaired baroreflex sensitivity, which can increase the risk of cardiovascular events, despite adequate blood pressure control.

## INTRODUCTION

Cerebrovascular diseases accounted for 99,233 deaths in Brazil in 2013 and occurred with a higher prevalence in adults aged 50 years old and older. Among these diseases, stroke was responsible for 140,207 hospitalizations between October 2013 and October 2014. During the same period, stroke caused 21,556 deaths, and health-related costs reached R$165,266,863.88 1. In the United States, it is estimated that 6.6 million people over the age of 20 years old have had a stroke 2.

Stroke is generally defined as an acute episode of neurological dysfunction that persists for at least 24 hours or until death and is caused by ischemia or hemorrhage 3. Cardiac disorders are common comorbidities in stroke patients and may further complicate the course of care in these patients 4. In this sense, cardiovascular autonomic dysfunction is often observed in acute post-stroke, as demonstrated by a reduction in heart rate variability (HRV), increased sympathetic modulation and decreased vagal modulation together with impaired baroreflex sensitivity, which is associated with early and late complications and mortality 5-10.

Regarding autonomic cardiovascular dysfunction in the post-stroke chronic phase, the existing data remain sparse and controversial. Korpelaine et al. 11 have suggested that fluctuations in HRV are reduced only in acute cerebral ischemic episodes and that these parameters return to normal over the following months. However, in a recent study conducted by our group, we observed decreases in vagal components of HRV in women with chronic stroke 12.

Thus, the aim of this study was to investigate cardiovascular autonomic function (heart rate variability, blood pressure variability and baroreflex sensitivity) in chronic post-ischemic stroke patients.

## METHODS

Ten patients with chronic post-ischemic stroke (5±1 years after stroke) were enrolled in The Neurological Physiotherapy Clinic of São Judas Tadeu University along with seven sex- and age-matched control individuals from the surrounding community. The inclusion criteria were as follows: age group, 40-65 years old, sedentary, non-smokers, at least 3 years since a first-time diagnosis of ischemic frontal-parietal stroke. The exclusion criteria were as follows: diabetes, recent cardiac event, renal failure, and patients using beta blockers. This study was conducted in accordance with Declaration of Helsinki and approved by the Ethics Committee of the Sao Judas Tadeu University (383.800), and all subjects signed an informed consent form before the start of the protocol.

Standard forms were used to collect data about clinical history and medications use. Measurements were performed in the morning, and subjects were instructed to refrain from consuming caffeinated and alcoholic beverages for at least 24 hours prior to starting the protocol.

Blood pressure (BP) was measured at rest by the auscultatory method with the patient in a sitting position. Continuous beat-to-beat noninvasive curves for BP (Finometer® FMS, Finapres Medical System, Holland) and electrocardiograms (ECG Amplifier, model 13-4615-64, Gould, USA) were recorded for 15 minutes using equipment coupled to a data acquisition system (WINDAQ, DataQ Instruments) at a sample rate of 1000 Hz. During this period, the participants remained in a supine position in a quiet room kept at a pleasant temperature (approximately 23°C).

The temporal series recorded for cardiac pulse interval (tachogram) and systolic blood pressure (systogram) were analyzed to obtain data for HRV and BP variability and baroreflex sensitivity. Each heart beat was identified by a specialized algorithm using a customized routine (MATLAB 6.0) that could automatically detect systolic and diastolic pressure wave events. The R-R interval was calculated as the difference between the beginning and end points of the cycle (t1-t0) 13. Power spectral density was obtained by the fast Fourier transformation using Welch's method with a Hanning window and 50% overlap as previously described 14.

In the time domain, the following variables were analyzed: SDNN (standard deviation of normal to normal (NN) intervals, which estimates total HRV), RMSSD (the square root of the mean of the sum of the squares of differences between adjacent NN intervals, which represents cardiac vagal modulation of HR) and Var-SAP (the variance of systolic arterial pressure, which represents variability in SAP). In the frequency domain, the following two main spectral bands were considered according to references in the literature: low-frequency (LF; 0.04 to 0.15 Hz), which contains information about both vagal and sympathetic cardiac modulation; and high-frequency (HF; 0.15 to 0.4 Hz), which indicates vagal cardiac modulation. The power density of each spectral component was calculated in normalized units. The normalized units were obtained by calculating the percentages of LF bands (LF ms^2^) and HF bands (HF ms^2^) with respect to the total power after subtracting the power of very low frequency components (VLF; frequencies of <0.04 Hz). We also calculated the LF/HF ratio, which represents sympathovagal balance 14,15. To assess blood pressure variability in the frequency domain, we estimated the values of LF-SAP (the power in the low-frequency range of the systolic arterial pressure, which represents vascular sympathetic modulation). In addition, baroreflex sensitivity was determined by evaluating the power spectrum of the R-R and SAP series and its decomposition into components and was inferred from the alpha index (the square root of the ratio of R-R LF ms^2^/LF-SAP mmHg^2^) 16,17.

Statistical analyses were performed using SPSS software (Version 20.0 for Windows; SPSS Inc., Chicago, USA). The Shapiro-Wilk test was used to assess the normality of the data and comparisons between groups were performed using Student's t-test for independent samples. The data are presented as the mean±standard error of the mean (SEM). *P* values <0.05 were considered significant.

## RESULTS

The demographic and clinical characteristics of the subjects are shown in Table 1. No statistically significant differences were observed between the stroke group (SG) and control group (CG). There were no significant differences in systolic or diastolic blood pressure or heart rate between the groups. The subjects in the SG had suffered a stroke 5±1 years prior to inclusion in this study.

Table 2 presents the results of HRV in the time and frequency domains. SDNN and VarNN, which represent total HRV, were lower in the SG than in the CG. RMSSD and the power of RR in the HF band, when expressed as percentage units, were lower in SG than in CG, indicating that cardiac parasympathetic modulation was lower in post-stroke patients, while sympathovagal balance (LF/HF ratio) was higher in SG than in CG.

**Figure 1:**
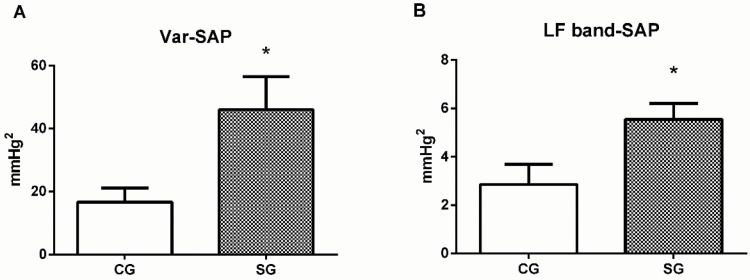
Blood pressure variability. (A) Variance in systolic arterial pressure (Var-SAP). (B) Low-frequency systolic arterial pressure (LF-SAP). CG: control group. SG: stroke group. Student's t test. *p<0.05 *vs.* CG.

**Figure 2:**
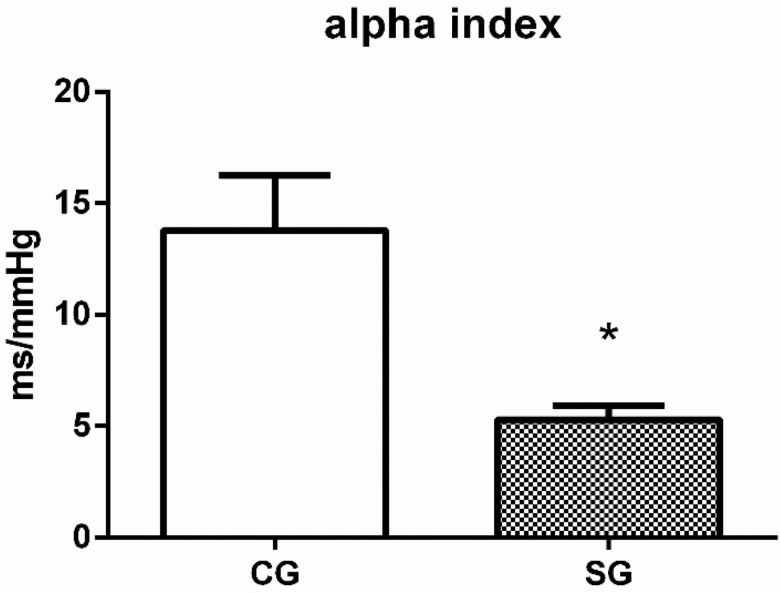
Baroreflex sensitivity (alpha index). CG: control group. SG: stroke group. Student's t test. *p<0.05 *vs.* CG.

Figure 1 shows the results of our analysis of SAP variability. The variance in SAP (46±10.5 *vs.* 16.7±4.5 mmHg) and the LF-SAP (5.6±0.6 *vs.* 2.9±0.8 mmHg^2^) were higher in SG than in CG. Baroreflex sensitivity, measured by alpha index, was lower in post-stroke patients than in controls (5.3±0.6 *vs*. 13.8±2.4 ms/mmHg), as shown in Figure 2.

## DISCUSSION

In our study, our subjects, at an average of 5 years after stroke, had lower HRV and cardiac vagal modulation. Moreover, our data are, to our knowledge, the first to demonstrate that chronic post-ischemic stroke patients presented increased systolic BPV and impaired baroreflex sensitivity, despite adequate control of BP values.

Previous studies have shown that abnormalities in cardiovascular autonomic modulation, measured by estimating HRV, may be viewed as an independent predictor of cardiovascular events and mortality 18,19. Our findings show that at five years post-stroke, HRV remains reduced as is accompanied by impaired parasympathetic modulation. Our group previously demonstrated similar results in chronic post-stroke women 12. Similarly, Dütsch et al. 20 found that parasympathetic cardiac modulation was reduced in patients at 30 months after stroke.

BP variability has been found to be an important predictor of both target organ damage 21,22 and cardiovascular and all-cause mortality 23. A candidate mechanism underlying organ damage due to BPV may be the activation of local angiotensin II and mineralocorticoid receptor systems, leading to chronic myocardial inflammation, which can result in cardiac hypertrophy and fibrosis 24. We observed that chronic post-stroke patients showed high systolic blood pressure variability (SBPV), despite adequate control of BP levels. The results of a systematic review suggested that greater short-term SBPV after acute stroke is associated with an increased risk of disability and mortality 25. In a recent study performed in patients with acute ischemic stroke, high mid-term BPV within 7 days of onset was associated with recurrent stroke and cardiovascular events during a 12-month follow-up period 26.

Increased short-term BPV may reflect alterations in the regulatory mechanisms associated with the autonomic nervous system, such as enhanced sympathetic drive and an impaired baroreflex function 21. Indeed, we observed higher sympathetic vascular modulation and lower baroreflex sensitivity in the SG than in the CG. Impaired baroreflex sensitivity has been found to be associated with an increased risk of cardiovascular events and mortality in several disorders, such as myocardial infarction 19,27, coronary surgery 28, heart failure 29, diabetes 30, and acute ischemic stroke 7. Unlike our findings, Dütsch et al. 20 detected no differences in sympathetic vascular modulation and baroreflex function in patients at 30 months after stroke.

We suggest that the motor disability triggered by stroke, which leads to physical deconditioning and a sedentary lifestyle, may represent a mechanism that is a candidate for an association with cardiovascular autonomic dysfunction in post-stroke subjects. In a previous study, we found that patients with chronic stroke presented lower maximal oxygen uptake than was observed in age-matched healthy individuals 12. The findings reported in the present study suggest that chronic stroke patients remain at high risk of cardiovascular events, including another stroke. Hence, autonomic nervous system modulation may be an important therapeutic target for chronic-post stroke management.

This study has some limitations, such as its small sample size and the use of antihypertensive drugs in the SG. Although we excluded patients who were taking beta blockers, another antihypertensive medication may have impacted cardiovascular autonomic modulation. Another limitation is the lack of a protocol for challenging the autonomic nervous system. Rodriguez et al. 31 evaluated alterations in HRV during orthostatic challenge at approximately 7 months post-stroke and found that the LF component of HRV when moving from a seated to a standing position was lower in the stroke group than in the control group, suggesting that cardiovascular autonomic dysfunction persists after recovery from stroke and that the cardiovascular response to standing may be impaired in these patients.

Despite the adequate control of BP levels, chronic post-ischemic stroke patients present lower heart rate variability, impaired cardiac parasympathetic modulation and baroreflex sensitivity as well as increased systolic BPV and vascular sympathetic modulation. These findings collectively indicate that even years after stroke, affected patients can present cardiovascular autonomic dysfunction that may be related to an increased risk of new cerebrovascular or cardiovascular events.

## AUTHOR CONTRIBUTIONS

Grilletti JV was responsible for the data acquisition, analysis and interpretation and conception and design of the study. Scapini KB was responsible for the data analysis and interpretation and drafting of the manuscript. Bernardes N and Spadari J were responsible for the data acquisition, analysis and interpretation. Bigongiari A and Mazuchi FA were responsible for the data analysis and interpretation. Caperuto EC and Sanches IC were responsible for the interpretation of the data and drafting of the manuscript. Rodrigues B was responsible for the interpretation of the data, design of the study, and drafting of the manuscript. De Angelis K was responsible for the conception and design of the study and drafting of the manuscript. All authors approved this version of the manuscript.

## Figures and Tables

**Table 1:** Participant characteristics.

	CG (n=7)	SG (n=10)
Age (years)	54±2	56±2
Gender	5 W, 2 M	7 W, 3 M
Height (cm)	168±1	164±2
Weight (kg)	65±4	64±4
SBP (mmHg)	115±3	123±6
DBP (mmHg)	77±1	82±6
HR (bpm)	70±3	72±5
Medications	None	ACE inhibitor, simvastatin, and acetylsalicylic acid

Values are expressed as the mean ±SEM; ACE: angiotensin-converting enzyme; CG: control group; DBP: diastolic blood pressure; HR: heart rate; M: men; SBP: systolic blood pressure; SG: stroke group; W: women.

**Table 2:** Heart rate variability in the time and frequency domains.

	CG (n=7)	SG (n=10)	*p*
SDNN (ms)	39.1±2.8	24.7±3.3*	0.013
VarNN (ms2)	835.07±87.55	219.54±45.2#	0.0007
RMSSD (ms)	28.5±2.4	18.9±2.0*	0.043
LF (%)	32.6±5.1	41.3±4.6	0.248
HF (%)	29.4±5.5	15.2±2.7*	0.048
LF/HF	1.2±0.2	2.8±0.3*	0.023

Values are expressed as the mean ±SEM; CG: control group; HF: high-frequency band of heart rate variability; LF: low-frequency band of heart rate variability; RMSSD: root mean square differences of successive R-R intervals; SDNN: standard deviation of normal to normal R-R intervals; SG: stroke group; VarNN: variance of normal to normal R-R intervals. *p<0.05 *vs.* CG; #*p*<0.01 *vs.* CG.
